# Low-cost Method for Obtaining Medical Rapid Prototyping Using Desktop 3D printing: A Novel Technique for Mandibular Reconstruction Planning

**DOI:** 10.4317/jced.54055

**Published:** 2017-09-01

**Authors:** Ignacio Velasco, Soheil Vahdani, Hector Ramos

**Affiliations:** 1 DDS, Fellow, Oral and Maxillofacial Surgery Department, Peking University Hospital of Stomatology, Beijing, China; 2 DDS, Resident, Oral and Maxillofacial Surgery Program, University of Puerto Rico/Medical Sciences Campus, San Juan, PR; 3 DMD, Professor, Oral and Maxillofacial Surgery Program, University of Puerto Rico/Medical Sciences Campus, San Juan, PR

## Abstract

**Background:**

Three-dimensional (3D) printing is relatively a new technology with clinical applications, which enable us to create rapid accurate prototype of the selected anatomic region, making it possible to plan complex surgery and pre-bend hardware for individual surgical cases. This study aimed to express our experience with the use of medical rapid prototype (MRP) of the maxillofacial region created by desktop 3D printer and its application in maxillofacial reconstructive surgeries.

**Material and Methods:**

Three patients with benign mandible tumors were included in this study after obtaining informed consent. All patient’s maxillofacial CT scan data was processed by segmentation and isolation software and mandible MRP was printed using our desktop 3D printer. These models were used for preoperative surgical planning and prebending of the reconstruction plate.

**Conclusions:**

MRP created by desktop 3D printer is a cost-efficient, quick and easily produced appliance for the planning of reconstructive surgery. It can contribute in patient orientation and helping them in a better understanding of their condition and proposed surgical treatment. It helps surgeons for pre-operative planning in the resection or reconstruction cases and represent an excellent tool in academic setting for residents training. The pre-bended reconstruction plate based on MRP, resulted in decreased surgery time, cost and anesthesia risks on the patients.

** Key words:**3D printing, medical modeling, rapid prototype, mandibular reconstruction, ameloblastoma.

## Introduction

The need for reconstruction of mandibular defects has been a continuous challenge faced by most of oral and maxillofacial surgeons. Benign or malignant tumors, osteomyelitis, trauma, osteoradionecrosis, and most recently medication-related osteonecrosis of the jaws are conditions that commonly result in significant continuity defects in the mandible (1). The aims for reconstruction are the maintenance of proper esthetics and symmetry of the face and the achievement of good functional result, thus preserving the form and the strength of the jaw and allowing future dental rehabilitation (2). Following mandibular resection, depending the time of reconstruction, it can be at the same time of resection (immediate), or delayed the popular reconstruction procedures includes vascularized or non-vascularized bone, distraction osteogenesis and titanium reconstruction plates (1,2).

The use of titanium plate for reconstruction of these defects is a gold standard in practice of reconstructive surgery (3). Shaping of the long titanium plates for mandibular reconstruction is not easy and it is time consuming process. Their intraoperative preparing leads to longer surgical operation and increases the cost of the intervention (4). The proper adaptation of the plate to the anatomical surface is essential for the success of this procedure.

Preoperative surgical planning has evolved in the last 20-years with the advances of medical imagenology. However, current imaging modalities are limited by being displayed on a 2D surface, such as a computer screen. But with the concomitant advance of rapid prototype technology in the engineering field has led to the production of models from computerized imaging (5). Medical rapid prototyping (MRP) is defined as the manufacture of dimensionally accurate physical models of human anatomy derived from medical image data using a variety of rapid prototyping technology (6). The source data for the construction of these models is principally high-resolution computed tomography (CT) scan, although MRI and ultrasound have also been used. MRP was first used in medicine by Mankovich et al. in the early 1990’s (7). There are several technologies for production of MRP anatomic models which includes: stereolithography (SLA), selective laser sintering, multiphase jet solidification and three-dimensional (3D) printing.

3D printing is a manufacturing method in which objects are made by the mechanism of fused deposition modelling (FDM) of a thermoplastic material in layers to produce a 3D object. Some 3D printers are similar to traditional inkjet printers; however, the end product differs in that a 3D object is produced. There are about two dozen 3D printing processes, which use varying printer technologies, speeds, and resolutions, and hundreds of materials. These technologies can build a 3D object in almost any shape imaginable as defined in a computer-aided design file (8). Medical applications for 3D printing are expanding rapidly and are expected to revolutionize health care.

Recent literature has shown that MRP models can be successfully used in the perioperative period for improving the predictability of treatment of maxillofacial defects secondary to traumatic or pathologic conditions it is widely accepted that utilization of MRP models offers many distinct advantages for improved patient care (9,10). These, includes diagnosis and treatment planning, and for patient education with direct visualization of anatomic structures. Models can be used for surgical guides and templates, as well as surgical rehearsal for training residents as well as experienced surgeons. One can easily design soft tissue incisions, surgical resection margins, assess bony defects for grafting, adaptation and prebending of reconstruction plates (11).

Historically, the technically challenging nature of 3D software and the high prices of early 3D printers usually meant that clinicians should require the services of an external company for the creation of MRP models. However, since the past decade with massification of desktop 3D printers and user friendly 3D software’s, it is possible to have this technology in our clinics today.

The purpose of this article is to introduce how to create a mandible MRP model by using a non-industrial desktop 3D printer with data obtained from CT imaging and open source/free 3D modeling software’s. We also present three cases of mandibular resection due to pathology in which we used MRP models for surgical planning and plate prebending.

## Material and Methods

**Figure 1:**
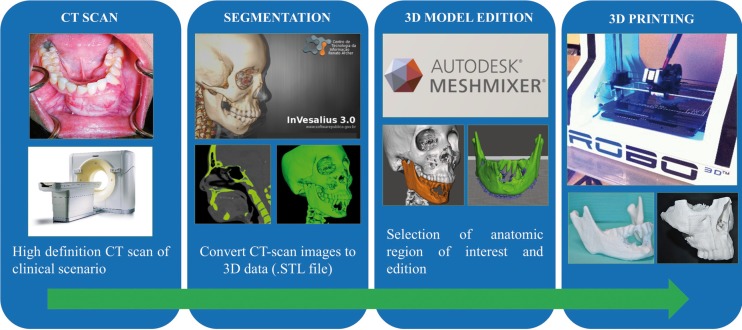
3D Medical Rapid Prototype Design Process.

We have treated three consecutive cases of mandibular tumors using MRP models after obtaining informed consent at the University of Puerto/Medical Sciences Campus for surgical planning and plate prebending. Diagnosis was obtained in all cases prior to the planification though incisional biopsy. Two cases were solid ameloblastoma and one case was an ossifying fibroma. The patient ages ranged from 34 to 41-year-old, all were females. The steps involved from imaging to the obtention of the 3D printed models are described in detail below and outlined in figure 1.

-3D Medical Rapid Prototype Design Process

CT imaging: High resolution CT scans were obtained following the same protocol. The protocol requires helical CT scan with field of view of 20-25 cm, slice thickness less than 1.25mm (equal to scan spacing) and gantry tilt 0°. It is important that the patient remains immobile during the study and the occlusal plane should be parallel to the gantry. In this way, we reduced the possible artifacts in the anatomic model. The data must be saved in uncompressed digital imaging and communications in medicine (DICOM) file type.

Segmentation: DICOM data was processed using the open source 3D imaging software InVesalius 3.0.0 version (Centre for Information Technology Renato Archer, Campinas, SP, Brazil). Using the tool for mask creation, bone mask is selected with 226-3071 Hounsfield unit range (can be increased in case of noisy result). For the creation of the 3D surface the bone mask was used as reference. Then the data was exported as stereolithography (STL) file. The process can take between 10-15 minutes depending the performance of the computer (computer with powerful CPU and graphic card is required).

-3D Model Edition, slicing and printing: The STL file was edited using the free software Autodesk Meshmixer® 2.9.1 version (Autodesk®, San Rafael, CA, USA) and using the selection/analysis tools the mandible was isolated and repaired to transform the complex geometry of the cancellous bone into a compatible model for printing, this process may take between 30-60 minutes depending the expertise of the operator. Finally using the open source MatterControl® 1.3.0 application the mandible mesh was prepared for the slicing and printing using the ROBO 3D R1 (ROBO 3D®, San Diego, CA, USA) with 1.75mm polylactid acid (PLA) filament (HATCHBOX, USA). The printing process depending the detail and size of the model can take between 4-7 hours. To calculate the MRP cost for each model, we need the final model weight in grams and multiply by U$ 0.022 which is an approximate retail cost for each gram of PLA 1.75 mm material.

**Figure 2:**
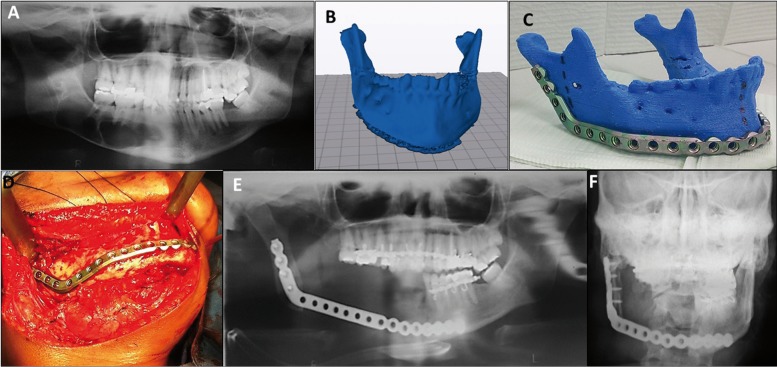
(A). Panorex x-ray showing a multilocular lesion, approximately 5.0 x 2.5 cm in size in the right mandible (B, C). MRP model created from patient DICOM data and reconstruction plate (2.7-mm mandible reconstruction plate (KLS Martin®, Jacksonville, FL, USA) prebended on its corresponding RMP. (D). Submandibular flap exposing the right mandible and tumor. (E, F) Postoperative panoramic and skull x-ray showing correct positioning of the reconstruction plate and mandibular symmetry.

-Case Presentation:

Case 1:

A 40-year-old female was referred to our clinic for evaluation of right perimandibular swelling with one year of evolution. Panorex and maxillofacial CT scan revealed a multilocular lesion that was approximately 6 x 2.5 cm in size in the right mandible, extending from the root of the canine to the mid-ramus (Fig. 2A). Incisional biopsy of the lesion resulted in solid ameloblastoma. An 8-cm mandibular resection with preservation of the right condyle was planned with titanium 2.7 mm reconstruction plate in the first stage and delayed reconstruction of the defect with iliac crest bone graft in a second stage. Before surgery using our protocol (Fig. 2B) the MRP was created and as reference the resection sites were planned and pre-bending of a reconstruction plate (2.7-mm mandible reconstruction plate (KLS Martin®, Jacksonville, FL, USA) was done (Fig. 2C). Intraoperatively, a submandibular flap was performed exposing the right mandible and tumor. The reconstruction plate was positioned without modifications and holes were drilled in healthy bone. Plate removal and segmental mandibulectomy was performed, followed by repositioning of the plate and fixation to the predrilled holes (Fig. 2D). The total surgery time was 3 hours and 55 minutes. A postoperative panoramic and skull x-ray showed correct positioning of the reconstruction plate and mandibular symmetry (Fig. 2E, F).

Case 2:

A 34-year-old female presented to our clinic with a large anterior mandibular solid ameloblastoma previously biopsied. Panoramic x-ray and maxillofacial CT scan revealed a multilocular lesion that was approximately 5 x 2 cm in size mainly in the symphysis region, extending from the root of the right canine to the left first molar. A 6-cm mandibular resection was planned with titanium 2.7-mm mandible reconstruction plate (KLS Martin®, Jacksonville, FL, USA) and immediate reconstruction of the defect with anterior iliac crest bone graft, allograft and INFUSE® (Bone Morphogenetic Protein, Medtronic®, Memphis, TN, USA). Before surgery using our protocol the RMP was created and the resection sites were planned and plate was prebended. Through intraoral approach the mandible and tumor were exposed. The reconstruction plate was positioned without modifications and holes were drilled in healthy bone. Plate removal and segmental mandibulectomy was performed, followed by repositioning of the plate and fixation to the predrilled holes. Titanium mesh (KLS Martin®, Jacksonville, FL, USA) was fixed to inferior border of the plate across the defect and filled with the bone graft previously obtained from anterior iliac crest and mixed with allograft and 8mg of INFUSE® (Bone Morphogenetic Protein, Medtronic®, Memphis, TN, USA). Oral mucosa was sutured watertight without tension to avoid wound dehiscence and graft exposure. The total surgery time was 5 hours and 30 minutes. A postoperative panoramic and skull x-ray showed correct positioning of the reconstruction plate and titanium mesh.

**Figure 3:**
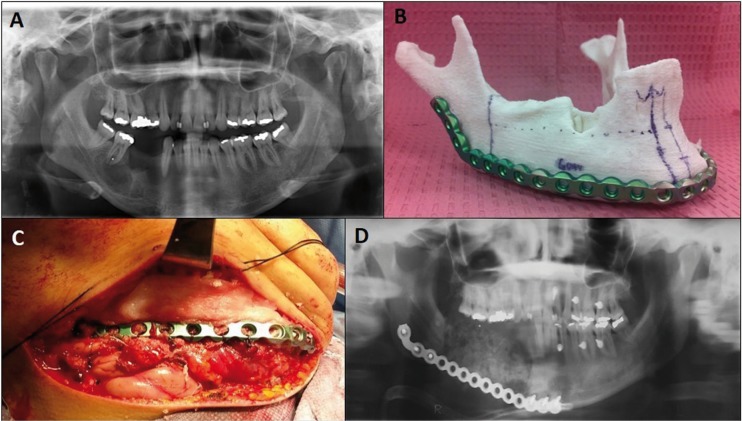
(A) Panorex revealing a mixed multilocular lesion that was approximately 6 x 2 cm in size mainly in the right mandibular body. (B) MRP model created from patient DICOM and its corresponding prebended plate. (C) Submandibular flap with plate and bone graft in place. (D) Postoperative panorex showing correct positioning of the reconstruction plate.

Case 3:

A 39-year-old female was referred to our clinic for evaluation of right perimandibular swelling with three year of evolution. Panorex and maxillofacial CT scan revealed a mixed multilocular lesion that was approximately 6 x 2 cm in size in the right mandible body, extending from the root of the canine to the first molar (Fig. 3A). Incisional biopsy of the lesion resulted in ossifying fibroma. A 8 cm mandibular resection with preservation of the right condyle was planned with titanium 2.7 mm reconstruction plate in the first stage and immediate reconstruction of the defect with iliac crest bone graft, allograft and 8mg of INFUSE® (Bone Morphogenetic Protein, Medtronic®, Memphis, TN, USA). Before surgery using our protocol (Fig. 3B) the MRP model was created and as reference the resection sites were planned and pre-bending of a reconstruction plate (2.7-mm mandible reconstruction plate (KLS Martin®, Jacksonville, FL, USA) was done (Fig. 3B). Intraoperatively, a submandibular flap was performed exposing the right mandible and tumor. The reconstruction plate was positioned without modifications and holes were drilled in healthy bone. Plate removal and partial mandibulectomy was performed, followed by repositioning of the plate and fixation to the predrilled holes (Fig. 3C). The total surgery time was 5 hours and 20 minutes. A postoperative panoramic and skull x-ray showed correct positioning of the reconstruction plate and mandibular symmetry (Fig. 3D).

## Results

In all cases MRP models were printed for surgical planning of mandibular resections and for plate prebending. The printing time of case 1 was 5 hours and 31 minutes and 4 hours and 38 minutes for case 2. MRP models were printed with 0.3 mm resolution. The cost of the PLA filament in both models was less than U$ 1. All MRP models were created precisely, the reconstruction plates were fixated without difficulties and intraoperative modification was not required ( Table 1). The three cases showed postoperative symmetry confirmed with clinical examination and imaging studies. No complications occurred during surgery.

**Table 1:**
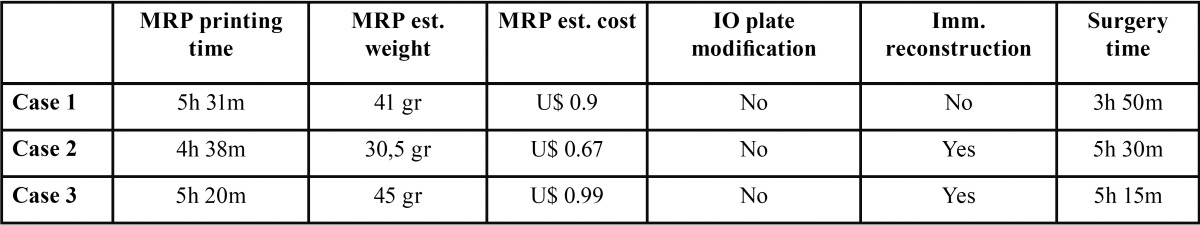
Results. Est: estimated, IO: intraoperative, Imm: immediate.

## Discussion

Regardless of the significant impact and media interest of the advancements with 3D printing technology. The incorporation of desktop 3D printing has not been adopted widely. One possible reason is the perception by clinicians is to believe that 3D printing is technically very complex. Therefore, the design and generation of these models is outsourced to an external company, which Increases the cost and time (12-14).

Two type of software are mainly required in MRP production; first, a “3D segmentation” software which translate the DICOM files from patient CT scan or Cone beam CT into a STL file, identifiable by 3D printers. Next ones are the “3D edition and slicing” software. There are plenty of 3D segmentation software available in market which are provided by software developers, such as 3D Slicer®, Visualization Toolkit (VTK)®, OsiriX® and Invesalius®. In our experience, we used InVesalius®, which is a free open-source, easy user interface and has the option to export the 3D model as a STL file. The 3D edition/slicing software digitally “slice” a STL file into layers suitable for 3D printing. This process can be readily performed using registered software’s that accompany the 3D printers or other available free/open source software at no extra cost and usually have a simple graphic user interface, such as Meshmixer® and Mattercontrol® for the ROBO 3D R1 printer.

The cost of early 3D printers, consisting mostly the SLA types, precluded widespread adoption of 3D printing in the initial years; however, the expiration of key patents surrounding SLA and FDM in the last decade has fueled a surge in the number of commercial developers leading to an increase in the availability and a significant reduction of the cost (14). Several affordable FDM 3D printers have entered the market since then, such as ROBO 3D R1®. In our group we use this printer due to its low cost (below U$ 1,000), high quality printing (0.1 mm as maximum resolution) and capability of generating printing with large designs (20.3 × 22.9 × 25.4 cm3). Currently, FDM 3D printers are the preferred option as a desktop application in medicine for their affordability and practicality (14).

In our experience MRP model created with desktop 3D printing, is a cost-efficient appliance for the surgical planning of mandibular resection and its reconstruction. Leaving aside the initial investment of a 3D printer and a last generation computer with a powerful graphic card, the cost of the material required to construct a mandible is not more than $1.00 US dollar with a variable printing time of 4-6 hours depending on its size and quality. With the protocol presented in this study any bone of the human body can be printed in a 1:1 scale. MRP models can be used as a patient’s anatomical replicas for patient educational purpose and explain them better the surgical treatment they are going to receive. Our patients described they had much better understanding about their condition and subsequent proposed surgical treatment. It is also an excellent academic tool in discussing the cases with our faculty members and helped us in concluding a better surgical approach which finally our patients will benefit from that.

Our team use MRP models created with 3D printing technology as anatomical template for mandibular reconstruction plate pre-bending and surgical planning in pathology cases. This novel low-cost technique has the advantage of reducing the metallic plate fatigue due to bending manipulation intraoperatively, and has a superior mandibular contour plate implementation. It´s application saves surgical time in the operating room, and is a major advantage not only for financial reasons, but also for patient’s health welfares by decreasing the exposure time to general anesthesia, the possible complications and the recovery period (1). Time savings in long surgeries, may represent 1 or 2 hours (1,15-20). Toro *et al.* reported reduction of the operating time of 1 to 1.5 hours when using MRP models and virtual reality surgical planning (21).

## Conclusions

In our experience, we demonstrated that MRP models production with 3D printing technology is a novel approach for reconstructive surgery plannification, providing functional low-cost anatomical models which are helpful in surgical planning and results in increasing surgery predictability. This novel approach results in reduction of surgery time, costs, patient morbidity and anesthesia risk which ultimately will increase patient satisfaction. We see the near future where the 3D printing will become a standard tool in academic institution and private practice helping surgeon, resident in improving their clinical and surgical skills. Its highly recommend oral and maxillofacial surgeons to get familiarized and start the application of 3-D printing technology in their day to day practice as an additional feature.
